# Supplementary material for: A Phase Ib Clinical Trial of Metformin and Chloroquine in Patients with IDH1-Mutated Solid Tumors
Source: Cancers (Basel). 2021 May 19;13(10):2474. doi: 10.3390/cancers13102474 (PMC8161333; doi:10.3390/cancers13102474)
Supplement: Supplementary file 1 [file cancers-13-02474-s001.zip › cancers-1193763-supplementary.pdf]

Supplementary Materials

A Phase Ib Clinical Trial of Metformin and Chloroquine in Patients with *IDH1*-Mutated Solid Tumors

Mohammed Khurshed, Remco J. Molenaar, Myra E. van Linde, Ron A. Mathôt, Eduard A. Struys, Tom van Wezel, Cornelis J. F. van Noorden, Heinz-Josef Klümpen, Judith V. M. G. Bovée and Johanna W. Wilmink

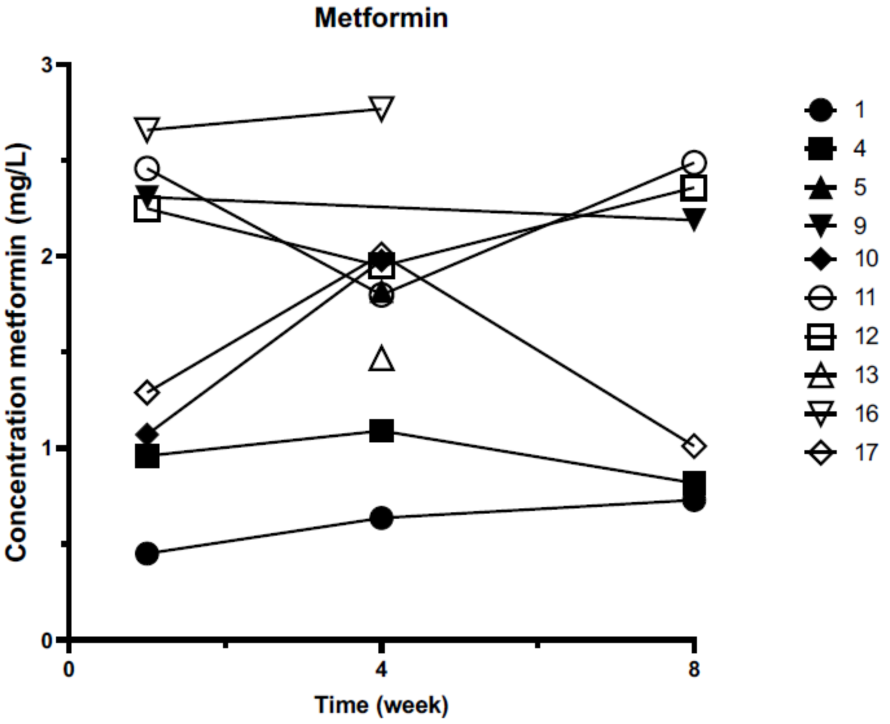

Figure S1. Serial metformin serum concentrations of patients in time. The plasma metformin concentration was comparable between single-agent administration (week 4) and co-administration with chloroquine (week 8).

© 2021 by the authors. Licensee MDPI, Basel, Switzerland. This article is an open access article distributed under the terms and conditions of the Creative Commons Attribution (CC BY) license (http://creativecommons.org/licenses/by/4.0/).
